# Sustainable and multifunctional mosquito pest management: a pull opportunity and a push advice

**DOI:** 10.1186/1756-3305-7-S1-O6

**Published:** 2014-04-01

**Authors:** B Seidel, F Allerberger, P Hufnagl, A Indra

**Affiliations:** 1 Technical Office of Ecology and Landscape Assessment, Persenbeug, Austria; 2 Department of Theoretical Biology, University of Vienna, Vienna, Austria; 3 Austrian Agency for Health and Food Safety, Vienna, Austria

## 

Since 2001, we have repeatedly detected pathogens during mosquito field studies in Austria (e. g. West-Nile-, Tahyna-, Usutu-virus and *Plasmodium *sp.). Recent surveillance discovered two invasive and two thermophilic mosquito species that were new for the Austrian fauna (*Aedes japonicus*, *Ae. albopictus*, *Anopheles hyrcanus *and *Culiseta longiareolata*). These species increase the possible spectrum of vector-borne communicable diseases in Austria. But until today Flaviviridae and Bunyaviridae have only been detected in context with the endemic mosquito species. Little attention was given to control pests and to develop sustainable, useful and affordable projects for controlling the dynamic of these indigenous mosquito populations. We emphasize the need for integration of mosquito control aspects, which up to now were ignored within contemporary Austrian river construction projects and we ask for a Europe wide consideration of mosquito control within the EU-Waterframework Directive (WFD); in particular we recommend mosquito control elements within fish ladders which are prescribed for water storage structures all over Europe. We also report on a material which allows the pulling of mosquitoes in the dimension of landscape management. We present our experience and emphasize the great advantage of actions working in connection with constructive river engineering.

